# Genetic and Cytological Analysis of a Novel Type of Low Temperature-Dependent Intrasubspecific Hybrid Weakness in Rice

**DOI:** 10.1371/journal.pone.0073886

**Published:** 2013-08-30

**Authors:** Chong-Yun Fu, Feng Wang, Bing-Rui Sun, Wu-Ge Liu, Jin-Hua Li, Ru-Fang Deng, Di-Lin Liu, Zhen-Rong Liu, Man-Shan Zhu, Yi-Long Liao, Jian-Wei Chen

**Affiliations:** 1 Rice Research Institute, Guangdong Academy of Agricultural Sciences, Guangzhou, P.R. China; 2 Guangdong Provincial Key Laboratory of New Technology in Rice Breeding, Guangzhou, P.R. China; 3 Public Laboratory, South China Botanical Garden, Chinese Academy of Sciences, Guangzhou, P.R. China; University of Massachusetts, United States of America

## Abstract

Hybrid weakness (HW) is an important postzygotic isolation which occurs in both intra- and inter-specific crosses. In this study, we described a novel low temperature-dependent intrasubspecific hybrid weakness in the F_1_ plants derived from the cross between two *indica* rice varieties Taifeng A and V1134. HW plants showed growth retardation, reduced panicle number and pale green leaves with chlorotic spots. Cytological assay showed that there were reduced cell numbers, larger intercellular spaces, thicker cell walls, and abnormal development of chloroplast and mitochondria in the mature leaves from HW F_1_ plants in comparison with that from both of the parental lines. Genetic analysis revealed that HW was controlled by two complementary dominant genes *Hw3* from V1134 and *Hw4* from Taifeng A. *Hw3* was mapped in a 136 kb interval between the markers Indel1118 and Indel1117 on chromosome 11, and *Hw4* was mapped in the region of about 15 cM between RM182 and RM505 on chromosome 7, respectively. RT-PCR analysis revealed that only *LOC_Os11g44310*, encoding a putative calmodulin-binding protein (*OsCaMBP*), differentially expressed among Taifeng A, V1134 and their HW F_1_. No recombinant was detected using the markers designed based on the sequence of *LOC_Os11g44310* in the BC_1_F_2_ (Taifeng A//Taifeng A/V1134) population. Hence, *LOC_Os11g44310* was probably the candidate gene of *Hw3*. Gene amplification suggested that *LOC_Os11g44310* was present in V1134 and absent in Taifeng A. BLAST search revealed that *LOC_Os11g44310* had one copy in the *japonica* genomic sequence of Nipponbare, and no homologous sequence in the *indica* reference sequence of 9311. Our results indicate that *Hw3* is a novel gene for inducing hybrid weakness in rice.

## Introduction

During evolution, ancestral species may diverge into several species that become genetically isolated from one another and develop a reduced capacity for hybridization due to pre- and postzygotic isolation [1], [2]. Prezygotic isolation inhibits the formation of zygotes, whereas postzygotic isolation occurs after the zygotes have formed. Postzygotic reproductive isolation is often expressed as embryonic lethality, seed inviability, weakness or sterility. The Bateson-Dobzhansky-Muller (BDM) model offers a theoretical explanation for postzygotic isolation [3], [4], [5]. This model presumes that hybrid incompatibility is caused by negative interactions between 2 or more unlinked genes of the nuclear genome or between nuclear and organellar genomes [6]. As one type of postzygotic barrier, hybrid weakness (HW) is defined as weak growth occurring in F_1_ hybrids derived from crosses between two normal parents and can be potentially explained by the BDM model.

HW has been found in a number of plant species, including *Oryza sativa* [7], *Arabidopsis thaliana* [8], and *Phaseolus vulgaris* [9], as well as in inter-specific crosses among *Nicotiana* species [10]. Some causal genes have recently been isolated and most cases of hybrid weakness or necrosis had a physiological response similar to pathogen attack, which suggested that the plant immune system could contribute to hybrid weakness or necrosis [11], [12]. For instance, the introgression of a resistance gene *Cf-2* from a wild relative into a domestic tomato led to hybrid necrosis [13]. In *Arabidopsis*, an allele of an NB-LRR disease resistance gene is both necessary and sufficient for the induction of hybrid necrosis by interaction with another unknown gene from the other parent [12]. In addition, Alcázar et al [14] found that an interacting QTL lying in a cluster of RPP1-like TIR-NB-LRR genes was associated with hybrid weakness and it requires the salicylic acid (SA) pathway activation induced by low temperature. These studies appeared to indicate that resistance genes might be linked with hybrid necrosis. Though transposons or retrotransposons might also be involved in hybrid incompatibility in *Arabidopsis* and *Drosophila*, HW in plants mainly resulted from negative consequences of immune system diversification and epistatic interactions among divergent immune components [12], [15], [16], which indicates the close relationship between hybrid incompatibility and evolution of plant immune system.

In China, hybrid rice is estimated to be planted on more than 50% of rice-growing land and it is credited with helping the country increase its rice yields. Hybrid weakness induced by low temperature will affect the application of heterosis in hybrid rice, to some extent, especially in the double-cropping rice areas of South China, although the frequency of hybrid weakness in rice is apparently much lower than that of intraspecific crosses in Arabidopsis [8]. Hybrid weakness in rice was reported more than four decades ago and its occurrence was sometimes conditioned by low temperature [17]. These hybrid weakness cases showed similar symptoms, including stunted growth, fewer tillers and abnormal root development. However, no gene for hybrid weakness has been cloned, and only several causal genes have been fine mapped to a relatively small genomic interval [18]–[21], especially the histological analysis on hybrid weakness was rarely involved in the previous studies, and the molecular mechanisms underlying hybrid weakness remain to be elucidated.

In this study, a novel intrasubspecific hybrid weakness was discovered in the F_1_ plants derived from the crosses between the *indica* varieties Taifeng A and some *indica* lines carrying blast resistance gene *Pi1*. We observed that these F_1_ plants showed the phenotype of hybrid weakness only in the early-cropping seasons with relatively low temperature at seedling stage, in Guangzhou, Guangdong province, South China. Temperature is known to influence disease resistance to pathogens. A high temperature very often inhibits disease resistance [22], which suggests low temperature should enhance disease resistance. Reduced growth of the plant is thought to be due to the high metabolic cost of maintaining activated resistance pathways [23]. Was the newly discovered hybrid weakness caused by enhanced resistance of *Pi1* gene at low temperatures? Here, we characterized the phenotype and analyzed the histological structures of the HW F_1_ plants derived from the cross between Taifeng A and V1134, conducted the genetic analysis and mapped the underlying genes.

## Materials and Methods

### Plant Materials

In the improvement of disease resistance for hybrid rice using MAS, all the F_1_ plants derived from the crosses between the elite *indica* line Taifeng A and the *indica* lines containing blast resistance gene *Pi1* displayed HW when they were grown in the early-cropping season with relatively low temperature (average daily temperature of 19.87°C from March to April based on thirty-three years’ historical data in Guangzhou, Guangzhou Province, South China). The F_1_ population of Taifeng A/V1134 showed hybrid weakness in the early growing season of 2009, and the F_2_ population were used to genetic analysis for the trait of hybrid weakness in the early-cropping season of 2010. One BC_1_F_2_ (Taifeng A/Taifeng A/V1134) populations including 952 individual plants was utilized for fine mapping the *Hw3* gene. Among of them, the line V1134 was derived from the cross between the elite line SH527 and the line GD7S/BL122 with blast resistance genes *Pi1* and *Pi2*. SH527 and GD7S are two *indica* varieties used widely in rice production in China. BL122 was bred by introgression of *Pi1* gene from the *japonica* cultivar LAC23 in West Africa and *Pi2* gene from Columbia variety 5173, into the i*ndica* variety Co39.

In the early-cropping season of 2010, Taifeng A and V1134 and their HW F_1_ progeny grown in the natural field conditions again was used to observe the cytological structure of the leaves and to screen for the candidate gene by RT-PCR.

### Phenotypic Characterization and Cytological Analysis

In order to characterize the phenotype of hybrid weakness, Taifeng A, V1134, and their HW F_1_ plants were grown in paddy field in the early-cropping season, 2011. Quantitative analysis of the agronomic traits including culm length, panicle length (PN), panicle number, and spikelet number per plant was conducted using 10 mature plants of Taifeng A, V1134, and their HW F_1_ line, respectively, grown in a paddy field.

Tissues from the same part of mature leaves were harvested from both parental lines and the HW F_1_ lines planted in the early-cropping season, 2011. The tissues were cut into approximately 1-mm^2^ pieces and fixed in 0.1 M phosphate buffer (pH 7.0) containing 2% glutaraldehyde. After 3 times wash with 0.1 M phosphate buffer, the leaf samples were post-fixed in 1% osmium tetroxide for 2 h. Next, the fixed leaf samples were dehydrated and embedded in flat molds using fresh resin. Semi-thin sections (1 µm) and ultra-thin sections (80 nm) were cut using a Leica-Ultracut S ultramicrotome and stained with toluidine blue and uranyl acetate, respectively. Five ultra-thin sections per sample were randomly selected to observe the cellular anatomy and chloroplast and mitochondrial structure using a transmission electron microscope (model JEM-1010; JEOL, Tokyo, Japan) operating at 100 KV. Five semi-thin sections per sample were used to observe the cell structure under a fluorescence microscope (AxioScope A1; ZEISS, German).

According to Van Wees’s protocol [24], the chlorotic leaves from the HW F_1_ and their parents were stained with 2.5 mg ml^−1^ trypan blue in a boiling water bath for 2 min, and cooled at room temperature for 1–24 h. The stained leaves were then destained in chloral hydrate solution at room temperature for 2–6 h. Finally, the samples were mounted in 70% glycerol.

### Measurement of Chlorophyll Contents (SPAD) and Photosynthesis

The five plants of the two parents Taifeng A and V1134 and HW F_1_ lines were randomly selected to measure the chlorophyll contents (SPAD value) using a SPAD-502 (Konica-Minolta, Osaka, Japan) and the photosynthetic rate using a portable photosynthesis system (LI-COR 6400, LI-COR Inc., Lincoln, NE, USA) at maximum tillering stage. The top three leaves from the main stems of every plant were used to survey the chlorophyll content, and the top, middle and base parts of each leaf were measured, respectively. The photosynthetic rate was measured at 10∶00–12∶00 am on a sunny day under the condition of CO_2_ concentration of 400 µmol mol^–1^and 800 Lux light intensity with the LI-COR LED (LI-COR Inc., Lincoln, NE, USA) irradiation source. The five top second leaves of the lines were measured.

### Temperature Sensitivity Tests

To analyze the sensitivity of the weakness phenotype to low temperatures, Taifeng A, V1134 and their F_1_ line were sown in experimental field in August (the late-cropping season with average daily temperature of 28.22°C), 2011. After 3 weeks, 40 plants from the F_1_ and their parents were randomly transplanted at 21°C and 32°C for 10 days in identical climate chambers (Sanyo, MLR-351H), respectively. Plants were observed every day for any aberrant phenotypes.

### Extraction of Genomic DNA

DNA was prepared using the modified hexadecyltrimethylammonium (CTAB) method. Fresh leaf material weighing ∼200 mg per sample was harvested from fully expanded leaves and placed in 1.5-ml microcentrifuge tubes. The samples were then frozen in liquid nitrogen and ground to fine powder. The ground samples were suspended in 750 µl of warm (65°C) CTAB buffer (2% [w/v] CTAB, 1.4 M NaCl, 20 mM EDTA, 100 mM Tris-HCl [pH 8.0]) and incubated at 65°C for 30 min. Next, 300 µl of chloroform: isoamyl alcohol (24∶1) was added to the mixture, and the microcentrifuge tubes were gently centrifuged for 5 min. The supernatants were transferred into fresh microcentrifuge tubes, and 800 µl of cold 95% ethanol were added to each tube. The samples were again centrifuged for 5 min, and the pellet obtained were washed twice with 70% ethanol and subsequently air-dried. The air-dried DNA were then dissolved in 500 µl of ddH_2_O and stored at –20°C prior to use.

### Mapping of Causal Gene for the HW

Ten normal plants and 10 HW plants from the BC_1_F_2_ populations derived from Taifeng A//Taifeng A/V1134 were used to create the recessive and dominant DNA pools, respectively, and DNA was extracted from the individual plants to perform bulked segregant analysis (BSA). Fine mapping of the HW genes was conducted with SSR, STS, and Indel markers. The primers for the STS and Indel markers were designed using Primer Premier 5.0 based on differences in the DNA sequences between *indica* and *japonica* rice, which are available at http://rice.genomics.org.cn (for *indica* 93-11) and http://www.rgp.dna.affrc.go.jp/(for *japonica* Nipponbare). The primer sequences and the amplified length of the developed DNA markers used in this study are listed in Table 2. PCR was performed in a reaction volume of 20 µl containing 10 ng of template DNA, 0.2 µM of each primer, 0.2 mM of each dNTP, 10 mM Tris-HCl (pH 8.3), 50 mM KCl, 1.5 mM MgCl_2_, and 1 U of *Taq* DNA polymerase. Amplification was carried out in a S1000 Thermocycler (Bio-Rad, USA) using the following conditions: 5 min at 94°C; followed by 35 cycles of 0.5 min at 94°C, 0.5 min at 55°C, and 0.5 min at 72°C; and a final extension of 5 min at 72°C. PCR products were separated in 3.0% agarose gels and stained with Gold View (Applygen Technologies Inc).

**Table 2:** The SPAD values and the photosynthetic rates for Taifeng A, V1134 and the HW F_1._

	SPAD values			Photo. Rate (µmol CO_2_ m^−2^s^−1^)
	Top 1st	Top 2nd	Top 3rd	
V1134	36.3±4.3a	44.3±2.54a	45.2±1.73a	22.41±0.99a
Taifeng A	36.6±4.27ab	42.1±2.15ab	43.2±1.74ab	20.18±0.95ab
HW F_1_	20.7±4.32c	36.2±1.94c	39.1±2.62c	13.87±0.61c

### RT-PCR Analysis

To confirm the candidate gene and examine its expression pattern, RT-PCR analysis was performed on the samples from the parental lines and F_1_ plants. The leaves were harvested from plants growing in the growth chamber (showing HW phenotype) and the paddy field. Total RNA was extracted from the leaves using SV Total RNA Isolation System (Promega). First-strand cDNA was synthesized from 1 µg of DNase I-treated RNA samples in a 20-µl reaction solution with random primers, using a ReverTra Ace-a-kit (TOYOBO). The PCR-amplified products were separated by electrophoresis through a 2% agarose gel and stained with Gold View. The *OsActin1* gene was used as the internal control for equal loading of cDNA template.

### Sequencing of the Candidate Gene

The full-length genomic DNA sequence of the candidate gene was divided into 7 segments to develop specific PCR primers. These primers were used to amplify genomic DNA from the two parental lines Taifeng A and V1134. PCR products were separated by electrophoresis,extracted and purified using a 3S Spin Agarose Gel DNA Purification Kit (Shanghai Biocolor Bioscience and Technology Company, China) for sequencing using specific primers with an ABI Prism 3730 XL DNA Analyzer (PE Applied Biosystems, USA). Sequence alignment was conducted with the BLAST network services (National Center for Biotechnology Information, NCBI and Beijing Genomics Institute, BGI).

## Results

### Characterization of the HW Phenotype

**Figure 1:**
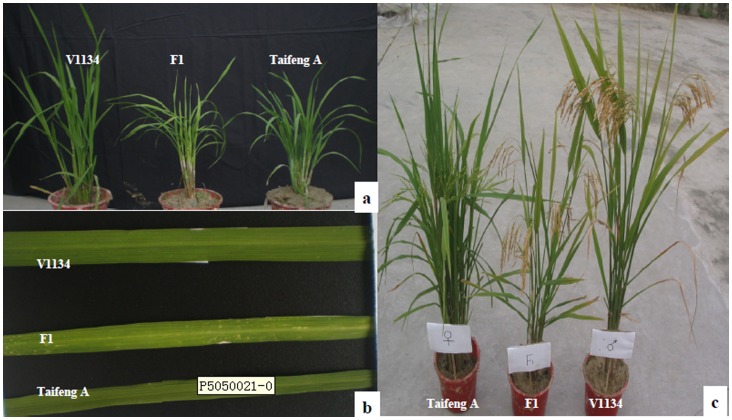
Phenotypic characterization of hybrid weakness in rice plants. (**a**) The morphology of the 40-day seedlings of Taifeng A, V1134 and their F_1_. (b) The leaf phenotypes of HW F_1_ and both parents’ 40-day seedlings. (c) The morphology of the HW F_1_ and their parents at mature stage.

In this study, the F_1_ population derived from the cross between two *indica* lines Taifeng A and V1134 showed a typical hybrid weakness phenotype. When grown at low temperature or in the early cropping season in South China, the F_1_ plants could be easily distinguished from both parental lines (Figure 1). The HW F_1_ plants showed short culms, fewer panicles, pale green leaves, and chlorotic spots in the mature leaves compared with their parents. We characterized the phenotypic traits of HW by measuring different agronomic traits including culm length (CL), panicle length (PL), panicle number (PN) and spikelet number (SN). As shown in Table 1, the panicle number, culm length, panicle length and spikelet numbers in F_1_ plants were 6.6, 61.2 cm, 21.2 cm and 142.4, respectively. They were significantly lower (p<0.05) than those of both parental lines, which were 7.8–7.9, 76.8–85.2 cm, 21.2–28.4 cm and 175.2–211 cm, respectively.

**Table 1:** Agronomic traits for Taifeng A, V1134 and their HW F_1_ plants.

	CL(cm)	PL(cm)	PN	SN
Taifeng A	76.8±0.837 a	21.2±0.748 a	9±1.412 a	175.2±13.36 a
V1134	85.2±1.304 b	28.4±1.517 b	7.8±0.837 b	211±12.39 b
HW F_1_	61.2±0.837 c	21.2±1.304 a	6.6±1.14 c	142.4±11.42 c

CL culm length, PL panicle length, PN panicle number, SN spikelet number.

### HW is Induced by Low Temperature

**Figure 2:**
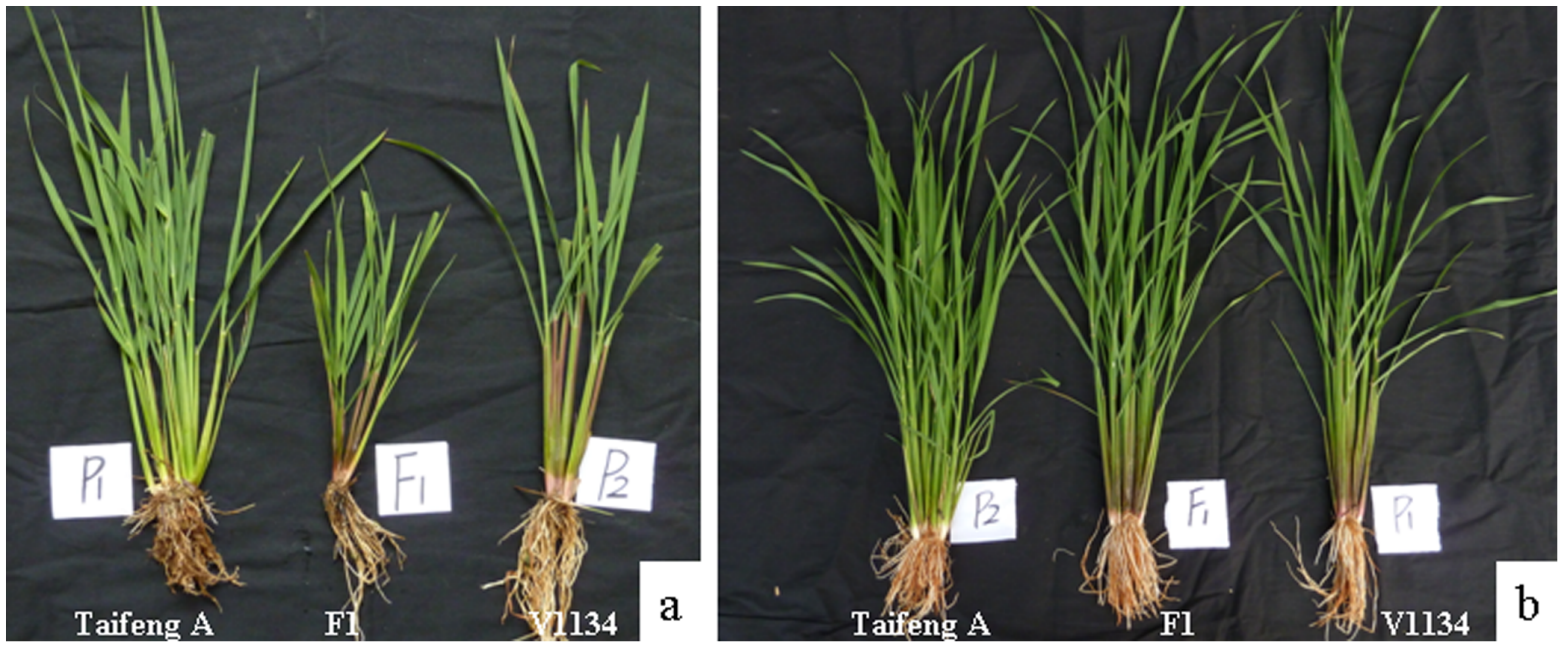
Phenotypic characterizations of the seedlings of Taifeng A, V1134 and their F_1_ grown under 21°C and 32°C. These 3-week-old seedlings grown in August, 2011 were treated under the conditions of 21°C and 32°C for ten days, respectively. (a) under 21°C. (b) under 32°C.

In early-cropping seasons (with relatively low temperatures at seedling stage, sown at the early of March) of four consecutive years (2009, 2010, 2011 and 2012), HW phenotype was observed in all F_1_ plants derived from the cross between Taifeng A and V1134. However, no HW phenotype was observed in late-growing seasons (with relatively high temperatures at seedling stage, sown at the end of July). We assumed that the occurrence of HW might be conditioned by temperatures. To test this hypothesis, the parental lines Taifeng A, V1134 and their F_1_ seeds were sown under natural field condition in July, 2011. After three weeks, 40 seedlings of the F_1_ and their parents were then transferred to versatile environmental test chambers (Sanyo, MLR-351H). Twenty of these plants were grown at 21°C, and the other 20 plants were grown at 32°C as control. After ten days’ treatment, the F_1_ seedlings growing at 21°C began to show symptoms of HW, including a pale green pigmentation in the primary leaves and growth retardation compared with their parents. But chlorotic spots in the primary leaves were not observed in the controlled growth condition (Figure 2). In contrast, the F_1_ seedlings growing at 32°C showed no HW phenotype at all, even with heterosis to some extent. These results corroborated our assumption that the observed HW was associated with low temperatures.

### Cytological Observation of the HW F_1_ Plants

**Figure 3:**
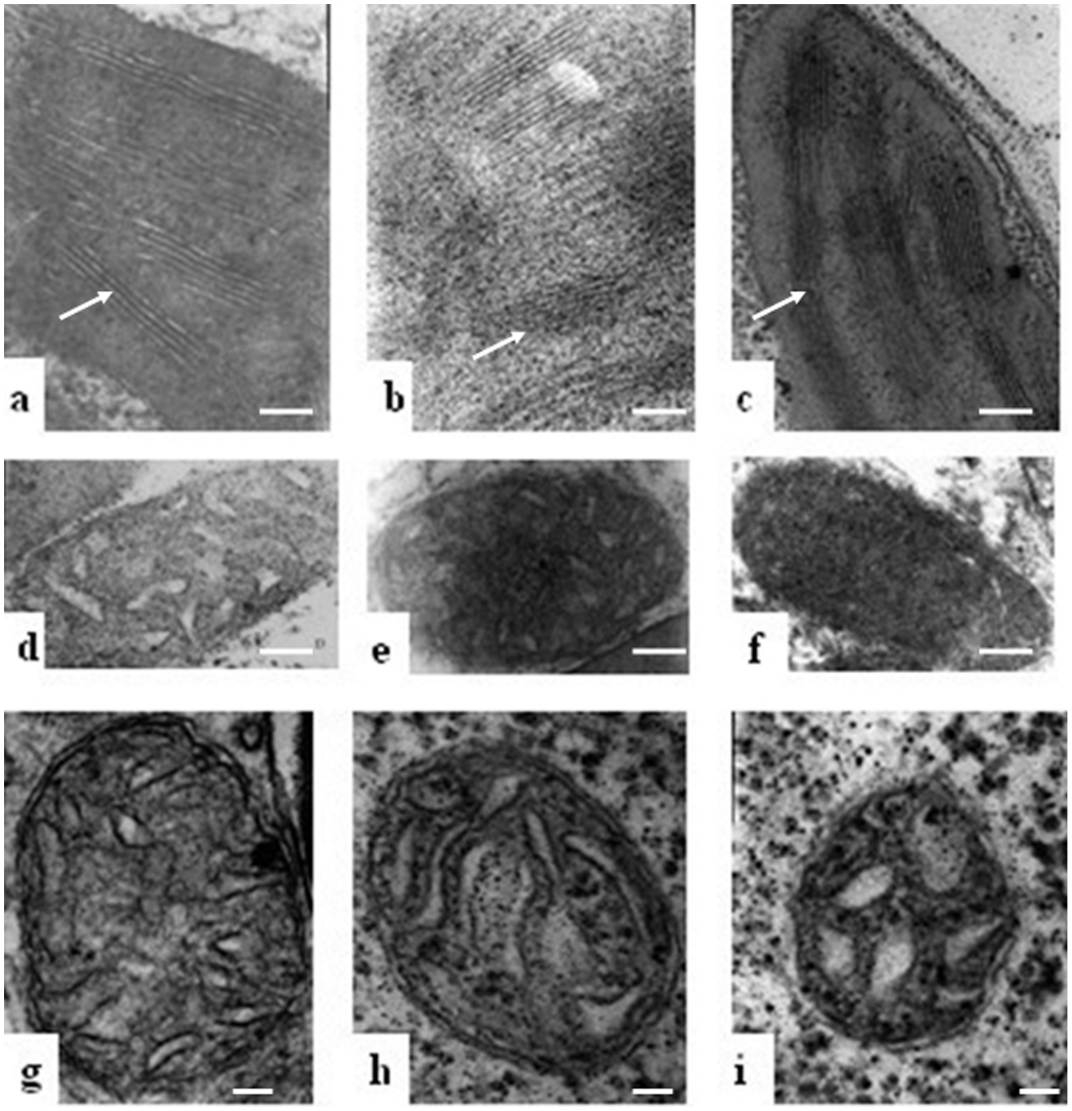
Transmission electron microscopic (TEM) observations of organelle structure of leaves and root tips in Taifeng A, V1134 and their F_1_ plants. The F_1_ and its parents were planted in the early-growing season with relative low temperatures for 5 weeks. (a and b) Chloroplast structures of the parents. (c) Chloroplast structure of the HW F_1_ plants. (d and e) Mitochondria structure of the leaves from the parent plants. (f) Mitochondria structure of leaves in the HW F_1_ plants. (g) and (h) Mitochondria structure of root tips from the parents. (i) Mitochondria structure of root tips from the HW F_1_ plants. Bars indicate 100 nm (a–f), 50 nm (g–i).

Previous studies have demonstrated that leaf discoloration mutants are related to chloroplast biogenesis [25]. To determine whether the pale green phenotype of the HW F_1_ plants was caused by a defect in chloroplast biogenesis, the chloroplast ultrastructure of leaves from HW F_1_ and their parents was analyzed by transmission electron microscopy (TEM). As shown in Figure 3a and b, abundant stroma and stacked grana thylakoids were observed in the chloroplasts of primary leaves from both parents. In contrast, no grana thylakoid was detected in the chloroplasts of the HW F_1_ plants. Only some lamellar structures were observed in these aberrant chloroplasts (Figure 3c). These results also further indicated that the grana formation in the leaves of HW plants was defective under low temperatures.

Growth retardation of HW plants under low temperatures could be associated with the aberrant metabolism of energy, which indicated that the defection of mitochondrial biogenesis was involved. We also compared the mitochondrial ultrastructure in the mesophyll cells by TEM. As a result, typical crista structure formed by the folding of the inner membrane of a mitochondrion was observed in the mitochondria of leaves from the parental lines (Figure 3d and e). In HW F_1_ leaves, however, the typical mitochondrial structure was not found. There were only mitochondrial precursor-like structures in mesophyll cells around the vascular bundles and they were quite obscure (Figure 3f).

To further verify the abnormal mitochondrial biogenesis in the other tissues of HW F_1_ plants, the mitochondrial structure from root tips cells was investigated by TEM. The mitochondrial volume in the cells of root tips from HW F_1_ plants was apparently smaller than those of their parents, and the folding of the inner membrane in these mitochondrial structures was mostly defective in the HW F_1_ plants (Figure 3g, h and i). These results indicated that mitochondrial biogenesis was affected in HW F_1_ plants.

**Figure 4:**
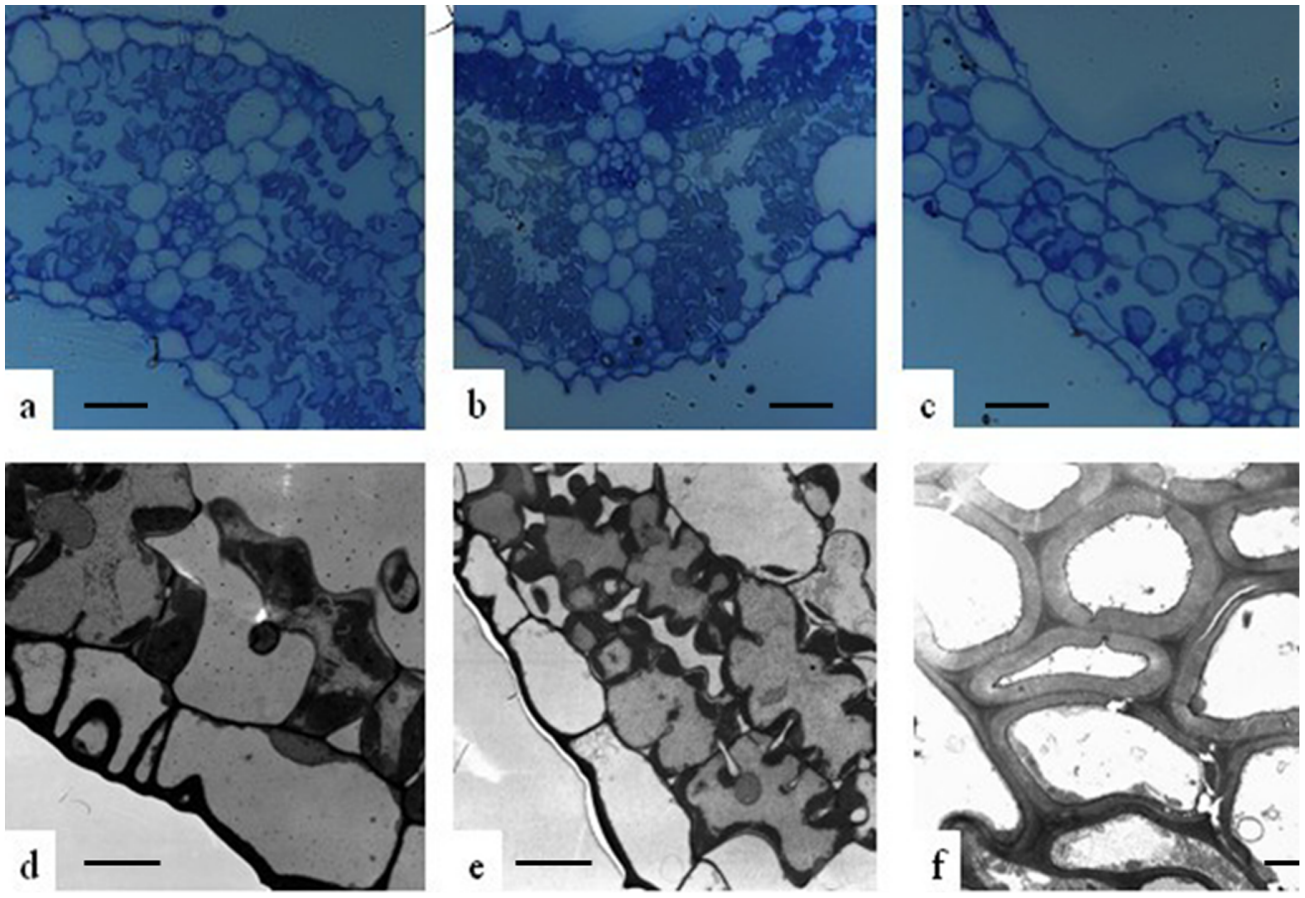
Cell characteristics of the leaves in Taifeng A, V1134 and their F_1_ plants. (a–c) Light microscopic observations of mesophyll cell structure of the HW F_1_ and their parents in transverse section. (a and b) Mesophyll cells of the parents (c) Mesophyll cells of the HW F_1_ plants. (d–f) Transmission electron microscopic (TEM) observations of cell wall structure of the HW F_1_ and their parents. (d and e) Cell wall of the parents. (f) Cell wall of the HW F_1_ plants. Bars indicate 20 µm (a, b and c), 5 µm (d and e), 2 µm (f).

The final size of a plant organ depends on both the number and size of its cells [26]. To investigate the cellular basis of the HW F_1_ plants, mesophyll cells were examined in the primary leaves from HW F_1_ plants and the parental lines using semi-thin plastic embedded sections. Compared to the parental lines, the size of mesophyll cell in HW F_1_ leaves did not significantly different. However, the number of mesophyll cells from HW F_1_ leaves was much lower and they were distantly distributed and loosely arranged with large intercellular spaces (Figure 4a, b and c), indicating that cell division was attenuated in the HW F_1_ plants. Interestingly, the cell walls of the HW F_1_ plants were remarkably thickened compared with those of their parents (Figure 4d, e and f).

**Figure 5:**
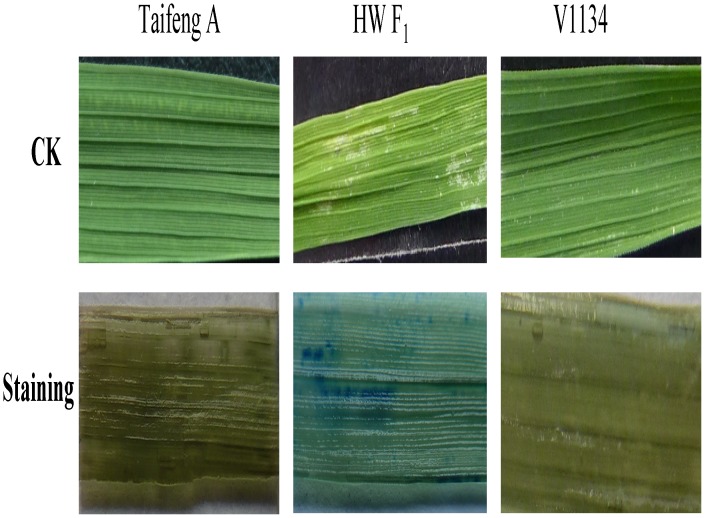
Cellular responses of the leaves from Taifeng A, V1134 and their HW F_1_ stained with trypan blue.

To further analyze the reasons for chlorotic spots occurring in the mature leaves of HW F_1_ plants, the leaves from both parents and their HW F_1_ were stained with trypan blue. Small and scattered blue spots were observed in the leaves of HW F_1_, but not occurred in the leaves of the parents (Figure 5), which suggested that the chlorotic spots in the leaves of HW F_1_ should be caused by cell death.

### Analysis of Chlorophyll Contents and Photosynthesis

Because the HW F_1_ plants showed pale green leaves and chlorotic spots in the leaves, and the chloroplast development was abnormal, the chlorophyll contents (SPAD values) and photosynthetic rate of the HW F_1_ was evaluated. The SPAD values and photosynthetic rate were listed in Table 2. The results showed that the SPAD values from the top first leaves to the top third leaves were gradually increased in all of the parental lines V1134, Taifeng A and their HW F_1_. In contrast, it was dramatically reduced in all the top three leaves of HW F_1_ compared to both the parents V1134 and Taifeng A. The photosynthetic rate of the HW F1 was 13.87 µmol CO_2_ m^−2^s^−1^, significantly lower than those (22.41 and 20.18 µmol CO_2_ m^−2^s^−1^) of the parents. The results suggested that the reduction of the chlorophyll content of the HW F_1_ plant began with the primary leaves, and its chloroplast development and chlorophyll biosynthesis were accordingly influenced.

### Genetic Segregation of HW Phenotype

To determine the inheritance pattern of the observed HW, 264 F_2_ plants derived from the cross between Taifeng A and V1134 (SH527//GD7S/BL122) were grown in the early cropping season with relatively low temperature. By defining the individuals with pale green leaves and chlorotic spots as weak plants, we investigated the number of individuals showing weak and normal growth in the F_2_ population. The segregation of the weak (HW) to normal (N) plants was 162 to 102, which fits the expected ratio of 9∶7 (**X** ^2^ = 2.805<3.841). The results suggested that two complementary dominant genes, here designated as *Hw3* and *Hw4,* respectively, genetically controlled the low temperature-conditioned HW. Some more severe weakness plants were observed in the F_2_ or BC_1_F_2_ (Taifeng A//Taifeng A/V1134) population (Figure S1), which further proved the two dominant gene model. Each parental line contributed one of the two dominant genes, namely, *Hw3* from V1134 and *Hw4* from Taifeng A, to the F_1_ progeny. In addition, we also observed that hybrid weakness occurred in the F_1_ plants derived from Taifeng A and BL122 in the early-cropping seasons (Figure S2), which suggests that *Hw3* gene should originate from BL122, the donor of *Pi1* gene.

### Mapping of the HW Genes *Hw3* and *Hw4*

Genetic analysis suggested that the HW was governed by two complementary dominant genes contributed by each parental line. To map the *Hw3* and *Hw4* genes from V1134 and Taifeng A, 360 SSR markers from the International Rice Genome Sequencing Project evenly distributed over the 12 chromosomes were screened for polymorphisms between Taifeng A and V1134 [27]. As a result, 29 SSR markers showed genetic polymorphism between the two parents.

**Figure 6:**
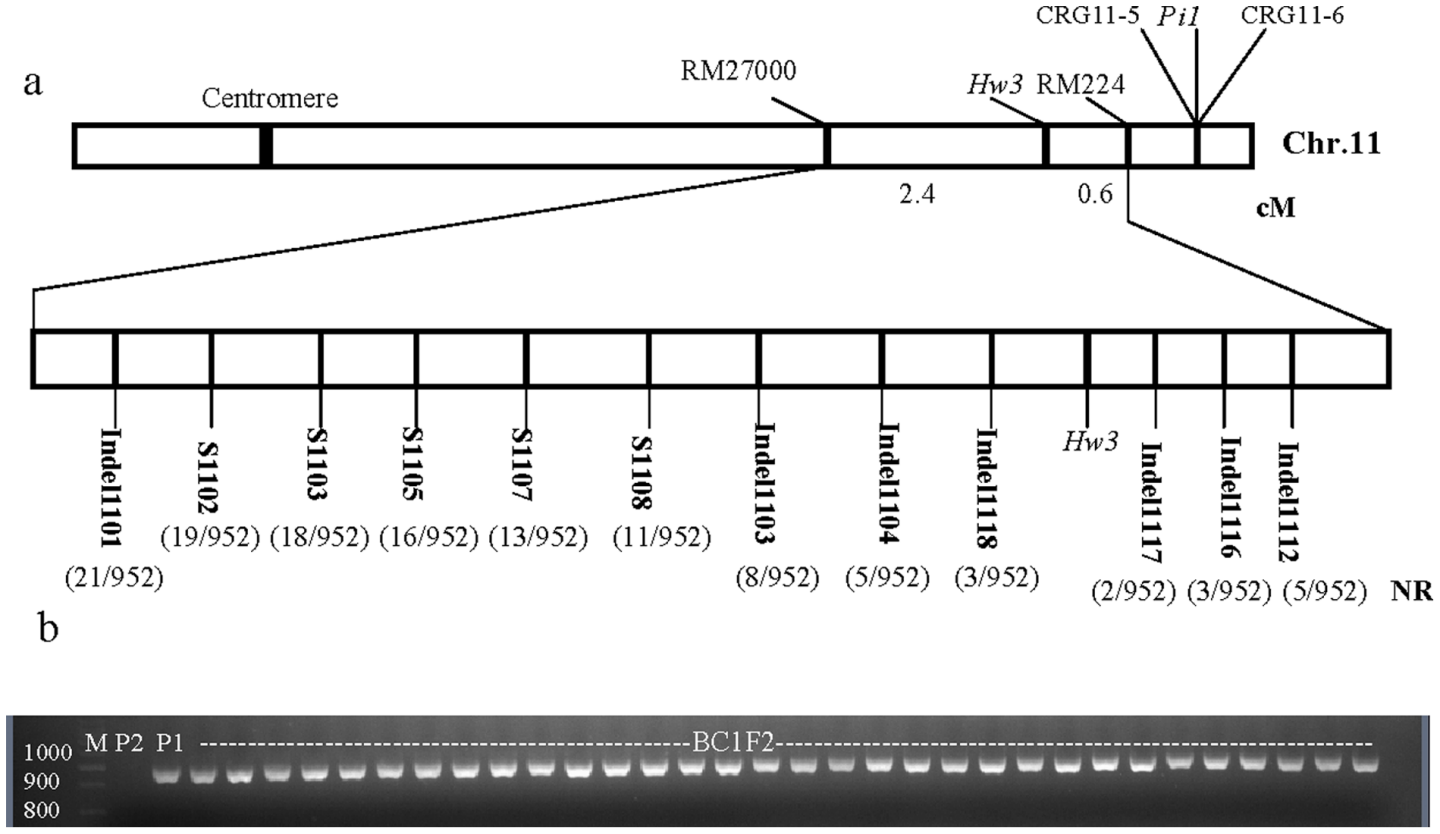
Linkage map of the *Hw3* gene and cosegregation analysis. (a) Linkage map of the *Hw3* gene. (b) Cosegregation analysis in the BC_1_F_2_ population of Taifeng A//Taifeng A/V1134 using the specific marker CBP2 (F: 5′ AGCATCTGGAAGCGGTTTTG 3′, R: 5′ CCATCTGCCTGTCACTATCATT 3′, the predicted size: 948 bp). P1: V1134. P2: Taifeng A. NR: the number of recombinants.

Linkage analysis of *Hw3* with the two polymorphic markers in the 292 BC_1_F_2_ (Taifeng A//Taifeng A/V1134)plants showed that the *Hw3* was located in between the markers RM27000 and RM224 on chromosome 11 with a distance of 2.4 and 0.6 cM, respectively. Because both genes *Hw3* and *Pi1* originated from BL122 and were linked to RM224.and *Pi1* consists of *Pi-5C* and *Pi-6C* [28], and two candidate resistance gene markers CRG11-5 of *Pi-5C* and CRG11-6 of *Pi-6C* could be anchored on Chr.11and located at the right side of RM224 based on the Nipponbare genomic sequence (http://www.gramene.org/Multi/blastview), the position of *Hw3* and *Pi1* could be clearly determined (Figure 6a).

To fine map *Hw3* gene, a population of 952 BC_1_F_2_ plants was used. Sixty SSR markers selected between RM27000 and RM224 did not display any polymorphisms between the two DNA pools. Hence, the DNA sequence of the region between RM27000 and RM224 was aligned at http://blast.ncbi.nlm.nih.gov/Blast.cgi based on the 93-11(*indica*) and Nipponbare (*japonica)* genomic databases, and 31 novel DNA markers (8 STS markers and 23 Indel markers) were developed in this region. Twelve markers showing polymorphisms between two DNA pools were used to genotype the 952 BC_1_F_2_ plants (Table S1, Figure 6). Eventually, the *Hw3* gene was delimited by the two flanking markers Indel1117 and Indel1118. Two and three recombinants were detected in the BC_1_ F_2_ population for Indel1117 and Indel1118, respectively. The physical distance between Indel1117 and Indel1118 was 136 kb using the Nipponbare (*O. sativa* ssp. *japonica*) genomic sequence as a reference (http://rice.plantbiology.msu.edu/).

On the other hand, *Hw4* from Taifeng A was mapped in the about 15 cM interval between SSR markers RM182 and RM505 on the long arm of chromosome 7 with the 190-plant BC_1_F_2_ (Taifeng A/V1134//V1134).

### Prediction of the Candidate Gene for *Hw3* and Sequence Analysis

**Figure 7:**
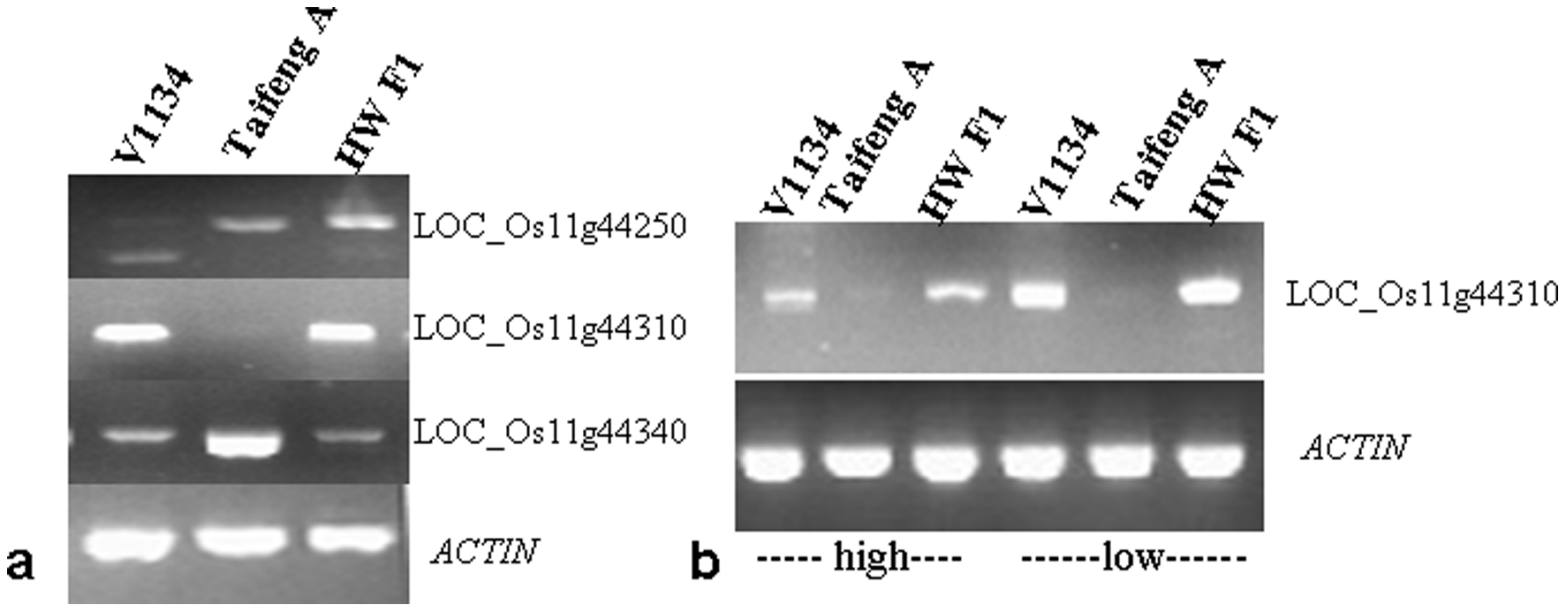
RT-PCR assays to screen the candidate gene causal to hybrid weakness in the candidate region and detect sensitivity to low temperatures in Taifeng A, V1134 and their F_1_ plants, and *OsActin1* gene used as the internal control. (a) Expression analysis of the annotated genes in the candidate region in Taifeng A, V1134 and their HW F_1_ plants. (b) Expression analysis of the candidate gene *LOC_Os11g44310* in Taifeng A, V1134 and their F_1_ plants after 10-day treatments of 21°C and 32°C temperature, respectively.

Because our mapping population was generated from a breeding program, the genetic differences between the two parental lines were small. It was very difficult to develop new polymorphic markers in the limited region where the target gene harbored. In order to predict the candidate genes for *Hw3*, we deployed a strategy of expression analysis using RT-PCR. To this end, the genes in the region were predicted according to the annotation databases (http://rice.plantbiology.msu.edu/and http://rapdb.dna.affrc.go.jp/). The region contains 18 annotated genes: 2 calmodulin-binding proteins, 1 protein kinase, 6 small expressed proteins, 3 hypothetical proteins, and 6 transposons or retrotransposons. The expressed hypothetical proteins in this region were relatively short, and the transposons or retrotransposons did not show transcriptional activation in general [15]. Thus, these short sequences and the transposable elements were excluded through the expression analysis. The cDNA primers for the remaining three predicted genes were designed with Primer Premier 5 based on the putative cDNA sequence of these genes in http://rice.plantbiology.msu.edu/ (Table S2). The transcript level of these genes was examined by RT-PCR using *OsActin1* as the internal control. The results indicated that only *LOC_Os11g44310*, which encoded a putative calmodulin-binding protein (CaMBP), displayed different expression among Taifeng A, V1134 and HW F_1_ (Figure 7a). It was expressed in V1134 and F_1_ plants at very similar level and was almost not detectable in Taifeng A. In addition, we compared the expression of *LOC_Os11g44310* in seedlings grown at 21°C and 32°C, respectively. The results showed that *LOC_Os11g44310* was expressed in V1134 and the F_1_ plants except Taifeng A in both treatments of 21°C and 32°C. Interestingly, it displayed much higher expression at 21°C than that at 32°C for both V1134 and F_1_ plants (Figure 7b).

**Figure 8:**
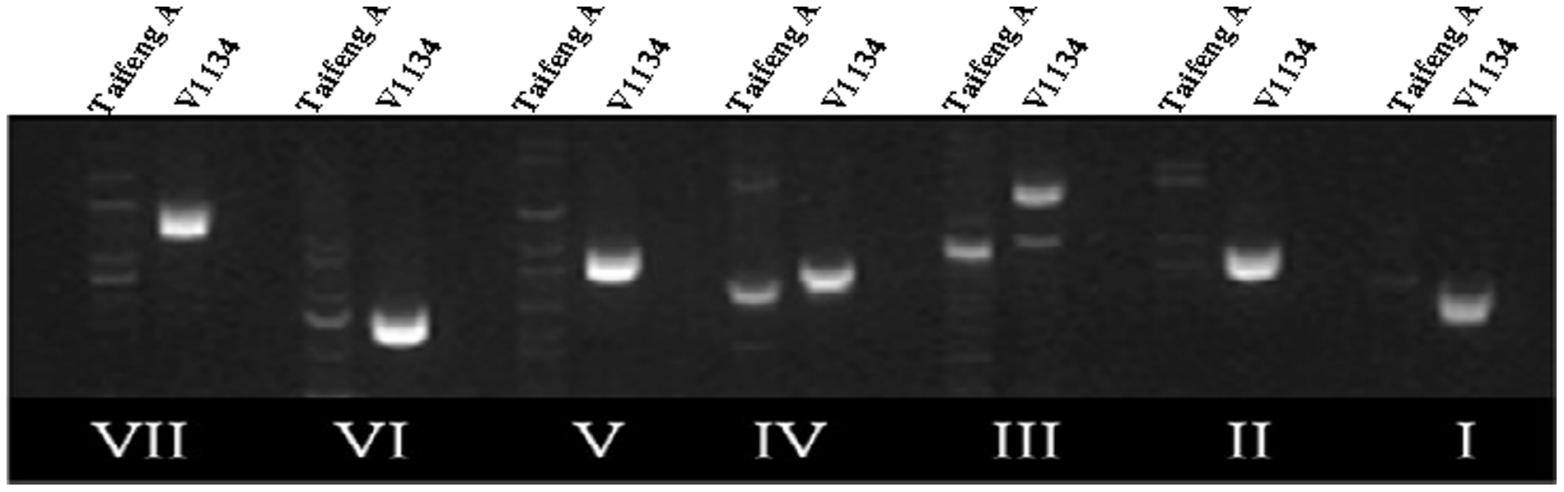
Amplification of the candidate gene between Taifeng A and V1134. (a) With the seven markers (CBP1-CBP7) specific to the seven divided segments of the candidate gene. I–VII: The markers of CBP1, CBP2……CBP7, respectively.

Due to no detection of transcript in Taifeng A, it was necessary to further verify whether *LOC_Os11g44310* was really lost in it, the complete genomic sequence of the candidate gene *LOC_Os11g44310* was amplified using 7 PCR reactions, which produced 7 over-lapping segments spanning the full-length genomic region. All the primers (CBP1∼CBP7) (Table S3) were designed based on the Nipponbare (*japonica*) reference sequence. All 7 segments were successfully amplified in the parental line V1134. However, only 2 (CBP3 and CBP4) of 7 primers could amplify the specific products showing different sizes between V1134 and Taifeng A (Figure 8a). The amplified products from Taifeng A were isolated and sequenced. BLAST analysis found that the sequence of the CBP3-amplified product from Taifeng A did not match the Nipponbare sequence, while the CBP4-amplified product from Taifeng A was aligned on chromosome 8. Therefore it could be concluded that the allele of *LOC_Os11g44310* was absent in Taifeng A. Hence, we speculated that *LOC_Os11g44310* could be the candidate gene for *Hw3.*

The specific marker CBP2 was used in cosegregation analysis to further determine the candidate gene for *Hw3*. Due to the absence of *LOC_Os11g44310* in Taifeng A, the specific marker is a dominant marker. The single-exchange recombinant plants can’t be distinguished from the no recombinant plant and the double-exchange recombinant plants can be discriminated from no recombinant plants when the dominant marker is used in cosegregation analysis. The new marker was used in PCR analysis for 716 weak individuals (dominant population) of the 952 BC_1_F_2_ population and no double-exchanged recombinant plant was detected (Figure 6). There was no recombinant plant observed using the new marker when 236 normal plants (recessive population) were deployed for cosegregation analysis. Since these normal plants showed the same bands to Taifeng A and *LOC_Os11g44310* was absent in Taifeng A, the related figure of electrophoresis was not shown here. Hence, the predicted gene *OsCaMBP* (*LOC_Os11g44310*) was considered as the candidate gene for *Hw3*.

**Figure 9:**
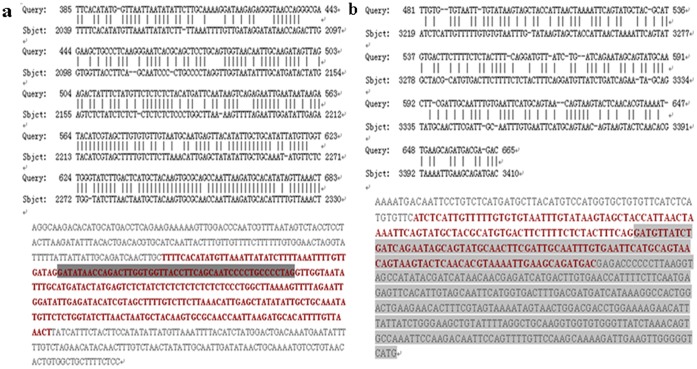
DNA sequence alignment between the amplified sequence in Taifeng A using the primers CBP3 and CBP4 and the reference sequence of Nipponbare. (A) Using the primer CBP3. (B) Using the primer CBP4. **Bold:** Location of selected alignment. **Highlighted Gray:** Location of the Exons.

In Nipponbare, the candidate gene *LOC_Os11g44310* consists of 8 exons and is predicted to encode a 542-amino acid protein based on the annotation databases (http://rice.plantbiology.msu.edu/). To analyze the structural characteristic of the candidate gene in V1134, these specific amplified products from the paternal line V1134 were cloned and sequenced. BLAST analysis indicated there was large sequence differentiation between V1134 and Nipponbare. For instance, the identities of sequences between the amplified products in the paternal line V1134 using the CBP3 and CBP4 primer pairs and the reference sequence of Nipponbare were only 89% and 84%, respectively. Further, BLAST analysis indicated that these large divergent sequences were at the intron-exon junctions (Figure 9), suggesting large differences could be found in the amino acid sequence encoded by the candidate gene and the putative amino acid sequence based on the reference Nipponbare sequence. BLAST analysis was performed on NCBI to investigate the copy number of the candidate gene *LOC_Os11g44310* in two rice subspecies (*indica* and *japonica*) (http://blast.ncbi.nlm.nih.gov/Blast.cgi). Only one copy of the candidate gene was found in the database of reference genome of *japonica* cultivars, and no homologous sequence of the candidate gene was found in the database of reference genome of *indica* cultivar.

## Discussion

### Linkage Drag and Hybrid Weakness

We found the hybrid weakness occurred in the F_1_ progenies derived from the crosses between Taifeng A and some improved rice lines carrying *Pi1* gene in the early-cropping seasons. Linkage analysis indicated that the SSR marker RM224 used to screen *Pi1* gene was linked to hybrid weakness. Is *Pi1* gene indeed the underlying gene of *Hw3*? Further linkage analysis and isolation of *Pi1* gene [28] found that *Hw3* and *Pi1* are linked to each other but not just the same gene. It is possible that *Hw3* from the donor BL122 was transferred along with *Pi1* into the recurrent parent. Although added backcrosses and large population could overcome the linkage drag, the linkage between *Hw3* and *Pi1* loci might be concealed when two complementary genes regulating the low temperature-dependent hybrid weakness did not occur in the same genome, or the breeding materials were grown in the high-temperature conditions of the late-cropping seasons. Several similar hybrid weakness or lethality cases were reported. The hybrid necrosis gene *Ne2* was found to tightly link to a leaf rust resistance gene *Lr13* in wheat [29]. The hybrid weakness gene *HWC2* in rice was closely linked to *Xa1* [19]. And several other resistance genes such as *Pikahei-1(t)* [30], *Pi39(t)* [31], *Xa2* [32] and *Gm7* [33] were also linked with the hybrid weakness *gene HWC2*. The wild flower *Mimulus guttatus* growing under copper tolerance condition displayed hybrid lethality which was induced by low temperature, and the genetic analysis showed that the hybrid lethality gene *Nec1* was closely linked to the copper tolerance gene *Tol1* [34].

In addition, *Hw3* existed in V1134, missed in Taifeng A and its candidate gene *LOC_Os11g44310* had large sequence variation in V1134 compared with the reference sequence of Nipponbare. Haplotype analysis of *HWC2* gene illustrated that its allele *hwc2-2* and *Xa1* originated from *indica* or tropical japonica and *Hwc2-1* gene diffused in temperate *japonica* [19]. *Ne2* locus has been differentiated into five alleles (*Ne2^w^*, *Ne2^mw^*, *Ne2^m^*, *Ne2^ms^*, and *Ne2^s^*) in wheat [35]. Tight linkage between hybrid ncompatibility and resistance loci in several independent cases seems to indicate that the coexistence between them could be a by-product of adaptive evolution.

### Low Temperature and Hybrid Weakness

Previous studies indicated that defense-related genes were up-regulated and photosynthesis-associated genes were down-regulated in hybrid weakness plants [36].In this study, the hybrid weakness was induced by low temperatures, and cytological observation indicated that there was large intercellular space, thickening of cell wall, chloroplast without grana thylakoids and mitochondrial without typical crista structure in HW F_1_ plants. The SPAD values of HW F_1_ plants gradually increased from the top first leaves to the top third leaves, but it was significantly less than both the parents, which indicated that chlorophyll biosynthesis was reduced in the HW F_1_ plants. And the photosynthetic rate of the HW F_1_ was significantly lower than those of the parents. Currently, several hybrid weakness or necrosis cases in some plants were previously reported to be temperature sensitive [8] and high temperature could rescue hybrid weakness [17]. Autoactivation of immune responses associated with Arabidopsis hybrid breakdown at low temperature has been described [8]. A number of CaM-binding proteins that are pathogen-inducible have been identified, suggesting that they may participate in the defense response [37], [38]. Cell death has a central role in innate immune responses in both plants and animals. The results of Trypan blue staining indicated that cell death existed in the leaves of the HW F_1._ *LOC_Os11g44310* was found to be remarkably up-regulated in Nipponbare upon the inoculation with *M. grisea* isolate FR13 (http://www.plexdb.org/modules/PD_browse/experiment_browser.php) [39]. These above results suggests *LOC_Os11g44310* and the hybrid weakness should be associated with defense response.We also found that the expression of the candidate gene *LOC_Os11g44310* was up-regulated at low temperature (21°C).

Low temperature significantly influences chloroplast development and chlorophyll biosynthesis [40], and the granal thylakoids were usually affected more than the intergranal ones under chilling temperature [41]. On the other hand, the mitochondria in the soybean axes were slightly diminished, and lacked internal structure at low temperatures [42]. Loss of grana thylakoids carrying many proteins in chloroplast and cristae in mitochondria might result in hybrid weakness by influencing photophosphorylation, and oxidative phosphorylation and reducing the generation of ATP. Although temperature was considered to affect the degree of hybrid necrosis by alerting thresholds for defense and cell death pathway triggering [43] and *LOC_Os11g44310* was involved in defense response. It is currently unknown how low temperature affects the chlorophyll synthesis, chloroplast development and the interaction between *Hw3* and *Hw4* to cause hybrid weakness.

### The Candidate Gene of *Hw3* Encodes a Putative Calmodulin-binding Protein

*Hw3*, one of the two complementary dominant genes controlling the hybrid weakness was fine mapped and the candidate gene was found to encode a putative calmodulin-binding protein. Calcium ions play an important role as a second messenger in eukaryotic cells. Calmodulin, a ubiquitous calcium-binding protein, regulates many diverse cellular functions by interacting with a wide range of calmodulin-binding proteins. The diverse CaMBPs in plants harbor various motifs and display functional diversity [44], [45], [46]. Several studies reported the involvement of CaMBP in cell division [47], [48], plant defense responses [37], [38] and organelle development [49], [50], [51], [52].

We found that cell division, cell wall and the development of chloroplasts and mitochondria was influenced under low temperatures in the HW F_1_ plants. These results indicated that the alteration of cell structure could be associated with the up-regulated expression of *OsCaMBP*. In addition, we did not find any typical stacked grana thylakoids in the chloroplasts of the HW F_1_ leaves. Grana thylakoids act as the main vector of pigments in plants, and the defection of grana thylakoids caused the reduction of pigment accumulation resulting in the occurrence of the pale leaves and lower chlorophyll contents (SPAD values). And only some mitochondrial precursor-like structures were observed around the vascular bundles. This indicated that the development of mitochondria was affected more substantially than that of chloroplasts in HW F_1_ plants, although we don’t know currently how it affects the development of chloroplast and mitochondria in the HW F_1_ plants. Bussemer et al [52] reported that AFG1L1, a calmodulin-binding AAA^+^-ATPase, was localized in both mitochondria and chloroplasts, and was potentially localized on the inner membrane on the side towards the mitochondrial matrix rather than on the chloroplast using the prediction software of NPS@ (PBIL, Lyon, France). And AFG1L1 was considered to be originally a mitochondrial protein and that the dual localization of AFG1L1 in chloroplasts and mitochondria is a newly acquired secondary trait, suggesting that this type of calmodulin-binding protein could have a greater influence on the biogenesis of mitochondria than on that of the chloroplasts.

### Origin of the Candidate Gene of *Hw3*

The results showed that the candidate gene of *Hw3* was present in V1134, but absent in *indica* cultivar Taifeng A. Further BLAST search indicates that it has only one copy in the reference sequence of *japonica* group and no homologous sequence of it in the reference sequence of *indica* group based on http://www.ncbi.nlm.nih.gov/, which suggests that the candidate gene should originate directly from BL122, the donor of the resistance genes *Pi1* and *Pi2* to blast and indirectly from the Libya *japonica* variety LAC23 with *Pi1*. In previous studies, all the HW cases appear in the interspecific rice hybrids. The action of certain genes with a foreign genetic background could be enhanced by other genes in a specific background [52]. The introgression of the foreign gene might break up the original coadapted genes, and the epistatic interactions between these genes that are harmless in their native genetic background could be enhanced under the specific conditions, which have highly deleterious consequences (the occurrence of hybrid weakness) for F_1_ hybrid progeny [8].

Overall, the molecular, cytological, physical and functional analysis of *Hw3* and the cloning of another complementary gene *Hw4* will further enhance the understanding of the mechanism underlying hybrid weakness and provide an opportunity to compare the similarities and differences in the regulation of the hybrid weakness phenomena for different species.

## Supporting Information

Figure S1 The more severe weakness plants in the BC_1_F_2_ population of Taifeng A//Taifeng A/V1134.
(TIF)

Figure S2 The weakness phenotype of the F_1_ plants derived from the cross between Taifeng A and BL122.
(TIF)

Table S1 The 12 newly developed polymorphic molecular markers in the interval between RM27000 and RM224 on Chromosome 11 for fine mapping of *Hw3*.
(DOC)

Table S2 The markers used in the RT-PCR analysis of three predicted genes in the candidate region.
(DOC)

Table S3 The 7 markers (CBP1-CBP7) designed based on the reference sequence of Nipponbare used to amplify the candidate gene *LOC_Os11g44310*.
(DOC)
