# Supplementary figures and images for: Genetic and Cytological Analysis of a Novel Type of Low Temperature-Dependent Intrasubspecific Hybrid Weakness in Rice
Source: PLoS One. 2013 Aug 30;8(8):e73886. doi: 10.1371/journal.pone.0073886 (PMC3758327; doi:10.1371/journal.pone.0073886)

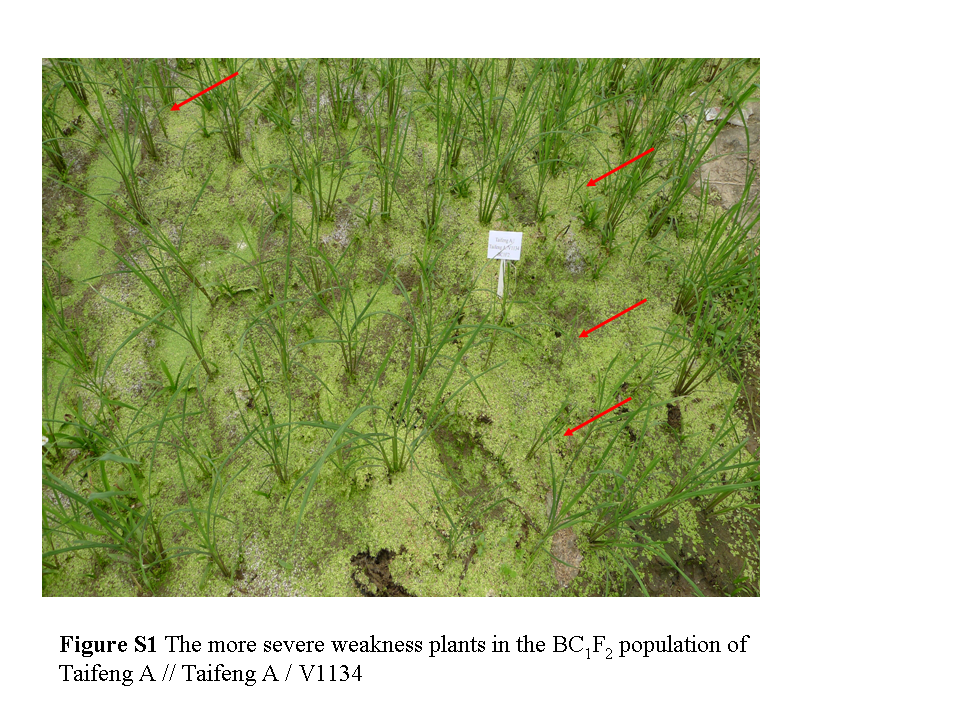

Supplement: Figure S1 — The more severe weakness plants in the BC1F2 population of Taifeng A//Taifeng A/V1134. (TIF) [file pone.0073886.s001.tif]

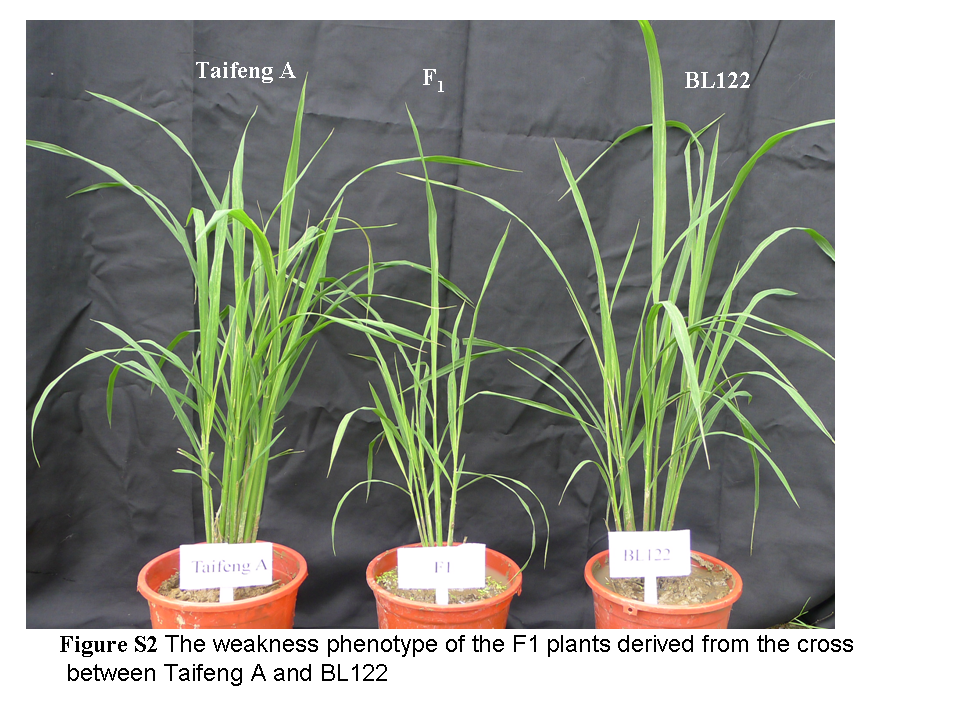

Supplement: Figure S2 — The weakness phenotype of the F1 plants derived from the cross between Taifeng A and BL122. (TIF) [file pone.0073886.s002.tif]
