# Supplementary material for: Genetic and Cytological Analysis of a Novel Type of Low Temperature-Dependent Intrasubspecific Hybrid Weakness in Rice
Source: PLoS One. 2013 Aug 30;8(8):e73886. doi: 10.1371/journal.pone.0073886 (PMC3758327; doi:10.1371/journal.pone.0073886)
Supplement: Table S1 — The 12 newly developed polymorphic molecular markers in the interval between RM27000 and RM224 on Chromosome 11 for fine mapping of Hw3. (DOC) [file pone.0073886.s003.doc]

**Table S1** The 12 newly developed polymorphic molecular markers in the interval between RM27000 and RM224 on Chromosome 11 for fine mapping of *Hw3*

| Marker | Predicted | Forward primer (5'–3) | Reverse primer (5'–3) |
| --- | --- | --- | --- |
|  | size (bp) |  |  |
| Indel 1101 | 290 | CAACATCTCGCAGGCACTCT | CTTGGAACCTTGGCAACTCT |
| STS1102 | 202 | TTTTATGCCGCTCTTGTGCT | ACGAGTGCGACTTAGGTTTCA |
| STS1103 | 477 | TTGGGCTGGGCAATAACTAC | CTGACTTCATCTCCGCTCCT |
| STS1105 | 399 | TACACCCAGATTCAGACGATG | AAGCCGCAATGACAACAGAG |
| STS1107 | 186 | TGGAGAACGGCTCGCTGGAT | GGAGGATGTTGGACGGCTTG |
| STS1108 | 152 | TGGGTGGCAGAAGGGTTTAT | ACCGTGAAGTATTCGGAGGC |
| Inde1 1102 | 322 | TTGAGTTGGCTGAAATGCTAA | AATCTTTGTGGAACCGTGTCT |
| Indel 1104 | 294 | GCCATTGACGGTGACTTTGA | ACAGTATCCCCGAACATCCTT |
| Indel 1112 | 173 | TATCGGTTCCTGACAATACC | AGACCCATCAACGAATACAG |
| Indel 1116 | 130 | TTCAAAGAGAGAGAATAGAAGCA | AAACCTTATCATCCCCGTAT |
| Indel 1117 | 187 | TTGGATGATAGCAGCAATAC | TTTGTTTAGATCCCACTGCT |
| Indel 1118 | 152 | GCTCTAGGACAGTTTGTTGG | TCATTCACAAAGCACAAAAG |
