# Supplementary material for: Genetic and Cytological Analysis of a Novel Type of Low Temperature-Dependent Intrasubspecific Hybrid Weakness in Rice
Source: PLoS One. 2013 Aug 30;8(8):e73886. doi: 10.1371/journal.pone.0073886 (PMC3758327; doi:10.1371/journal.pone.0073886)
Supplement: Table S2 — The markers used in the RT-PCR analysis of three predicted genes in the candidate region. (DOC) [file pone.0073886.s004.doc]

**Table S2** The markers used in the RT-PCR analysis of three predicted genes in the candidate region

| Predicted ORFs | predicted | Forward primers (5’–3’) | Reverse primers (5’–3’) |
| --- | --- | --- | --- |
|  | Size (bp) |  |  |
| LOC_Os11g44250 | 280 | CGATGACGGCTCAGCAGAAC | GGGACGAGGAGGAGATAGGA |
| LOC_Os11g44310 | 303 | ACGCCAGCAAACTTCTTCAG | TCGTCCGTGGATGGATTACC |
| LOC_Os11g44340 | 470 | GTGATTGTGCCAAATAGGTCC | GCTGCTACTGCGTGCTTCTC |
