# Supplementary material for: Genetic and Cytological Analysis of a Novel Type of Low Temperature-Dependent Intrasubspecific Hybrid Weakness in Rice
Source: PLoS One. 2013 Aug 30;8(8):e73886. doi: 10.1371/journal.pone.0073886 (PMC3758327; doi:10.1371/journal.pone.0073886)
Supplement: Table S3 — The 7 markers (CBP1-CBP7) designed based on the reference sequence of Nipponbare used to amplify the candidate gene LOC_Os11g44310. (DOC) [file pone.0073886.s005.doc]

**Table S3** The 7 markers (CBP1-CBP7) designed based on the reference sequence of Nipponbare used to amplify the candidate gene *LOC_Os11g44310*

| Marker | Predicted | Forward primer (5'–3) | Reverse primer (5'–3) |
| --- | --- | --- | --- |
|  | Size (bp) |  |  |
| CBP1 | 771 | ATAGGTGGATGGGCTGGATT | CTAACACGGTTTTGGTTTTGG |
| CBP2 | 948 | AGCATCTGGAAGCGGTTTTG | CCATCTGCCTGTCACTATCATT |
| CBP3 | 1434 | CCAACAAATGATAGTGACAGGC | AGGCACCACCACAAGGAAC |
| CBP4 | 881 | TGTTCACCGAAGGTTCCAGC | TTCTTTTCCAGGTCGTCCAG |
| CBP5 | 948 | GCCACTGGACTGAAGAACACT | AAAGAAGCAATCTCAACCGC |
| CBP6 | 691 | CAGAGGCGGTTGAGATTGCT | CTGAAGAAGTTTGCTGGCGT |
| CBP7 | 1305 | ACGCCAGCAAACTTCTTCAG | CATAGCCCTTTCGCTCCTTC |
